# Multidrug resistant tuberculosis: prevalence and risk factors in districts of metema and west armachiho, Northwest Ethiopia

**DOI:** 10.1186/s12879-015-1202-7

**Published:** 2015-10-26

**Authors:** Feleke Mekonnen, Belay Tessema, Feleke Moges, Aschalew Gelaw, Setegn Eshetie, Gemechu Kumera

**Affiliations:** Management Science for Health (MSH), HEAL-TB, Gondar, Ethiopia; Department of Microbiology, School of Biomedical and Laboratory sciences, College of Medicine and Health Sciences, University of Gondar, Gondar, P O. box: 196, Ethiopia; Department of Public Health, College of Medicine and Health Sciences, Debre Markos University, Debre Markos, Ethiopia

**Keywords:** Tuberculosis, MDR-TB, Risk factors

## Abstract

**Background:**

Multi drug resistant tuberculosis (MDR-TB) is an emerging challenge for TB control programs globally. According to World health organization, 2012 report Ethiopia stands 15^th^ out of the 27 high priority countries in the world and 3^rd^ in Africa. Updated knowledge of the magnitude of MDR-TB is so substantial to allocate resources, and to address prevention and control measures. Therefore, the aim of this study was to assess the prevalence of MDR-TB and associated risk factors in West Armachiho and Metema districts of North Gondar.

**Methods:**

A cross-sectional study was conducted in West Armachiho and Metema districts between February 01 and June 25, 2014. A total of 124 consecutive smear positive pulmonary tuberculosis patients were included in the study. Socio-demographic and possible risk factor data were collected using a semi-structured questionnaire. Drug susceptibility testing was first performed for rifampicin using GeneXpert MTB/RIF. For those rifampicin resistant strains, drug susceptibility testing was performed for both isoniazid and rifampicin to identify MDR-TB using the proportional method on LJ media. Data were analyzed using statistical Package SPSS version 20; binary logistic regression was used to assess the association. *P*-values < 0.05 were considered as statistically significant.

**Results:**

Of 124 smear-positive pulmonary TB patients, 117 (94.4 %) were susceptible to Rifampicin, while 7 (5.7 %) were confirmed to be resistant to Rifampicin and Isoniazid. The overall prevalence of MDR-TB was 5.7 % (2.3 % among new cases and 13.9 % among previously treated cases). History of previous treatment (OR = 7, *P* = 0.025) was significantly associated risk factor for MDR-TB.

**Conclusion:**

The overall prevalence of MDR-TB was 5.7 % among cases at five health centers and a history of previous treatment was found to be a risk factor for being infected by an MDR-TB strain. Therefore, maximizing early case detection and treatment, strengthening TB infection control activities and proper implementation of DOTS are recommended to reduce the burden of MDR-TB.

## Background

Tuberculosis (TB) remains a major global health problem. In 2012, World health organization (WHO) estimated 8.6 million people developed TB and 1.3 million died from the disease [1]. Even more, according WHO 2014 report, the morbidity and mortality rate were increased by 400,000 and 200,000 cases, respectively, with reference to the previous WHO report [2]. Besides both reports presented that the mortality of the disease was predominantly observed in human immunodeficiency virus (HIV) co-infection, thus 320,000 (2012) and 360,000 (2013) patients were died due to HIV co-infection. Tuberculosis compound by the spread of multidrug resistant (MDR) strains becomes a prime global concern, 450,000 and 480,000 multidrug resistant tuberculosis (MDR-TB) cases were reported in 2012 and 2013 respectively. Based on WHO 2014 report, the prevalence of MDR-TB among new and previously treated cases was 3.5 % and 20.5 % respectively. These estimates are essentially unchanged from 2012 [1, 2].

Emerging and spread of drug resistance TB has encountered as a great challenge in Africa region, Sub-Saharan Africa in particular. Information on the extent of MDR-TB from Africa region is very limited, probably due to poor laboratory facilities, poor surveillance mechanisms and reporting procedures, outdated databases and sub-optimal coverage of the infrequent surveys. Sub-Saharan Africa stands the burden of both very high TB incidence and the highest HIV prevalence rates in the world, and represents 14 % of the global burden of new MDR-TB cases [3].

Moreover, on the basis of WHO 2012 report, Ethiopia stands 15^th^ out of the 27 high priority countries in the world and 3^rd^ in Africa following South Africa and Nigeria with (1600 and 480) among new and retreatment cases respectively were MDR-TB cases [4]. According to the drug resistance survey conducted nationwide, the prevalence of MDR-TB was; 1.6 % in newly diagnosed TB and 11.8 % among previously treated TB cases. The country’s burden of MDR-TB in 2009 was estimated to be 1500 (870–2600) and 420 (230–740) among new and re-treatment cases respectively. Despite being a huge global threat, access to treatment is very limited with only 10 % of the estimated MDR-TB cases among notified TB cases in 2009 in the high MDR-TB countries and 11 % globally were enrolled in treatment [5, 6].

In West Armachiho and Metema districts, information regarding TB treatment interrupters, relapses, and failure cases were not well documented. According to the national comprehensive TB/HIV and Tuberculosis & Leprosy guideline these are criteria for suspicion of MDR-TB. Moreover, as to our knowledge, there are only limited data regarding MDR-TB in this particular study area. Therefore, current knowledge on the prevalence of MDR is so substantial to provide useful information on the implementation of standard chemotherapy regimens designed and recommended by WHO for tuberculosis patients who have or have not been treated previously.

Moreover, drug resistance rate can also serve as a useful parameter in the evaluation of the quality of current and past chemotherapy program i.e. direct observed treatment, short course (DOTS). Therefore, understanding the drug susceptibility patterns of *M. tuberculosis* (MTB) is very crucial to treat patients, to decide health priorities, to allocate resources, to monitor the emergency of resistance for planning effective use of anti-TB drugs, to generate knowledge for health workers working in the study area as well as will serve as a preliminary information for health programmers to give special attention and design a package in the national TB control program that addresses such areas where hundred to two hundred thousands of people are employed in huge farms for the production of crops.

## Methods

### Study area, study design, participants and data collection

A cross-sectional study was conducted in West Armachiho and Metema districts from Feburary 01 to June 25, 2014. A total of 124 smear positive patients were consecutively enrolled through convenient sampling technique. From those patients we gathered; socio-demographic characteristics (gender, age, residence, religion, occupation, marital status, income, ethnicity and education status) and possible risk factors (HIV, smoking, TB contact history, diabetes, fasting, history of prison, BCG vaccination status). Besides from each patient, standard volumes of sputum sample were collected after patients had given instructions accordingly.

### Sputum decontamination, isolation, identification & drug susceptibility testing

Smear positive sputum samples were re-confirmed using Gene X-pert and decontamination and further homogenization were done according to Petroff’s method. Isolates were identified by using typical colony characteristics on Lowenstein-Jensen (LJ) media and standard biochemical tests. Gene x-pert machine was used to assess rifampicin (RIF) resistant strains, after a while RIF resistant MTB isolates were tested for isoniazid (INH) and RIF using the indirect proportional method on LJ medium. The proportion method calculates the proportion of resistant bacilli present in a strain. Two appropriate dilution of the bacilli, 10^−2^ and 10^−4^dilutions (undiluted = 10^6^ to 10^8^ CFU/ml), were inoculated on drug-containing and drug-free media. Below a proportion (critical proportion = 1 %), the strain was classified as sensitive; otherwise classified as resistant. Patient’s HIV status was collected from the TB unit register at TB clinic of respective health facilities. Quality control was done for gene x-pert (sample processing and probe check) and LJ medium (standard strains of MTB H37Rv-ATCC27294).

### Data analysis

Data were entered and analyzed using SPSS version 20.0. Bivariate logistic regression analysis was used to assess the association. *P*-values < 0.05 were considered as statistically significant.

### Ethical consideration

Ethical clearance was obtained from the School of Biomedical and Laboratory sciences, University of Gondar. Written permission was obtained from North Gondar Zone Health Department to West Armachiho and Metema Woreda Health Offices and respective health centers. Study participants were recruited after getting written consent.

## Results

A total of 124 smear positive tuberculosis patients were included from five different heath centers in West Armachiho and Metema districts. The health centers were Abderafi, Abirihajira, Metema Yohannes, Gendewuha and Metema Hospital.

A majority, 80 (64.5 %) of the study participants were males, the mean and median age of the study subjects were 32 and 29 years respectively. Their age ranges from16–75 years. Nearly half, 46 (48.1 %) were in the age range of 26–35 year, while 37 (29.8 %) were below 25 years old. Of the 124 study subjects, 66 (53.2 %) were urban inhabitants and 59 (47.6 %) were farmers/day laborers. The majority, 116 (93.5 %) of the study subjects were Christians by religion while the rest 8 (6.5 %) were Muslims. More than half, 64 (51.6 %) were illiterate. More than half of new and previously treated cases were males (Table 1).

**Table 1:** Socio-demographic characteristics of TB patients West Armachiho and Metema districts, Northwest Ethiopia February 01 to June 25, 2014

Variable	Total TB cases (*N* = 124)	New active TB cases (*N* = 88)	Retreatment TB cases (*N* = 36)
Age group in years			
≤25	37 (29.8 %)	31 (35.2)	6 (16.7)
26–35	57 (46 %)	38 (43.2)	19 (52.8)
36–45	16 (12.9)	11 (12.5)	5 (13.9)
≥46	14 (11.3)	8 (9.1)	6 (16.7)
Gender			
Male	80 (64.5)	53 (60.2)	27 (75)
Female	44 (35.5)	35 (39.8)	9 (25)
Resident			
Urban	66 (53.2)	47 (53.4)	19 (52.8)
Rural	58 (46.2)	41 (46.6)	17 (47.2)
Occupation:			
Farmers and day laborers	59 (47.6)	41 (46.6)	18 (50)
House wife	28 (22.6)	21 (23.9)	7 (19.4)
Government employee	8 (6.5)	8 (9.1)	0
Merchant	16 (12.9)	13 (14.8)	3 (8.3)
Driver	5 (4)	1 (1.1)	4 (11.1)
Student	8 (6.5)	4 (4.5)	4 (11.1)
Income/month:			
<500 birr	39 (31.5)	29 (33)	10 (27.8)
500 birr - 999 birr	55 (44.4)	39 (44.3)	16 (44.4)
≥1000 birr	25 (20.2)	16 (18.2)	9 (25)
No means of income	5 (4)	4 (4.5)	1 (2.8)
Religion:			
Christian	116 (93.5)	82 (93.2)	34 (94.4)
Muslim	8 (6.5)	6 (6.8)	2 (5.6)
Ethnicity:			
Amhara	102 (82.3)	77 (87.5)	25 (69.4)
Tigre	22 (17.7)	11 (12.5)	11 (30.6)
Educational status:			
Illiterate	64 (51.6)	42 (47.7)	22 (61.1)
Primary school	36 (29.0)	26 (29.5)	10 (27.8)
Secondary school	17 (13.7)	14 (15.9)	3 (8.3)
Diploma and above	7 (5.6)	6 (6.8)	1 (2.8)
Marital status:			
Married	62 (50)	44 (50)	18 (50)
Un married	45 (36.3)	35 (39.8)	10 (27.8)
Widowed	5 (4)	4 (4.5 %)	1 (2.8)
Divorced	12 (9.7)	5 (5.7)	7 (19.4)

The proportion of smear positive tuberculosis cases in each health facilities were as follows: Abderafi 49 (39.5 %), Metema Hospital, 34 (29.8 %), Abirihajira 21 (12.9 %), Metema Yohannes 16 (12.9 %) and Gendewuha 4 (3.2 %).

### Prevalence of Multi- drug resistant tuberculosis (MDR-TB)

Sputum samples of the 124 smear positive tuberculosis patients were tested for MDR-TB by using Gene-Xpert MTB/RIF technique and conventional solid culture, The overall prevalence of MDR-TB was 7 (5.6 %, 95 % CI; 2.4–10.5 %) and prevalence of MDR-TB among new smear positive TB cases was 2 (2.3 %, 95 % CI; 0–5.9 %) and among previously treated smear positive TB cases 5 (13.9 %, 95 % CI; 2.9–25.7 %). Of the 26 who were reported to have been cured with prior treatment, 1 (3.8 %) and from10 who were reported to have failed or defaulted from prior treatment, 4 (40 %) were relapsed MDR-TB cases. Among 41 patients whose occupation was farmer/daily laborer, 2 (4.9 %) had primary MDR-TB; of 47 whose occupation was other than farmer/day laborer none (0.0 %) had primary MDR-TB. The majority of confirmed MDR-TB subjects were males 6 (85.7 %) and two of the confirmed MDR cases were co-infected with HIV (28.6 %) while, the other five were sero-negative.

Among the five health facilities, MDR-TB cases were obtained from Abderafi health center and Metema District Hospital. Three MDR-TB cases (one from new smear positive study subjects and two from retreatment cases) were identified from Abderafi health center. The other four MDR-TB cases (one from new smear positive study subjects and three from retreatment cases) were identified from Metema hospital.

### Rifampicin resistant Non-MDR-TB

Sputum samples of 124 smear positive pulmonary TB cases were processed using Gene-X pert MTB/RIF for detection of MTB and identification of RIF resistant strain. Of these only seven smears positive cases were found to be RIF resistant. Sputum samples of RIF resistant cases were further diagnosed with LJ medium for growth of MTB and detection of INH resistance as well as confirmation of RIF resistant strain. The result showed RIF resistant non-MDR-TB isolates were not observed from all RIF resistant isolates that were detected by Gene-X pert MTB/RIF for drug susceptibility testing. All seven RIF resistant isolates were found to be MDR-TB cases.

### Risk factors for MDR-TB

The relationship between individual exposure variables and MDR-TB status is shown in Table 2. Association between potential exposure variables and MDR-TB were analyzed. Socio-demographic determinants such as age, sex, residence, occupation, income, religion, fasting, ethnicity, educational status, marital status, and factors such as contact history, history of imprisonment, number of rooms in the house, family number in the household, rooms for sleeping, number of windows and use of substances like cigarette smoking and other clinical characteristics such as, diabetes, history of previous anti-TB treatment, outcome of previous treatment, BCG vaccination, HIV status, history of taking illegal anti-TB treatment were assessed.

**Table 2:** Factors associated with the MDR-TB status among Pulmonary TB cases, West Armachiho and Metema districts, Northwest Ethiopia, February 01 to June 25, 2014

Variable	MDR-TB		Crude OR	*P*-value
	Positive (*N* = 7)	Negative (*N* = 117)		
Age group				
≤25	1	36	2.77 (0.16, 47.56)	0.483
26–35	4	53	1.02 (0.11,9.90)	0.987
36–45	1	15	1.15 (0.07,20.34)	0.922
≥46	1	13	1	
Gender				
Male	6	79	3.49 (0.41, 29.9 4)	0.255
Female	1	43	1	
Resident				
Rural	4	54	1.56 (0.33, 7.26)	0.574
Urban	3	63	1	
Occupation:				
Farmer and day laborers	4	55	1.50 (0.32, 7.01)	0.604
Other	3	62	1	
Ethnicity:				
Tigre	3	19	3.87 (0.80,18.70)	0.092
Amhara	4	98	1	
Educational status:				
Illiterate	2	62	5.17 (0.41, 65.68)	0.206
Primary school	3	33	1.83 (0.16,20.71)	0.624
Secondary school	1	16	2.67 (0.14,49.76)	0.511
Diploma and above	1	6	1	
Fasting				
Yes	3	73	0.45 (0.10, 2.12)	0.313
No	4	44	1	
History of smoking				
Yes	2	33	1.02 (0.19, 5.51)	0.983
No	5	84	1	
BCG vaccination				
Yes	1	27	0.71 (0.13, 3.89)	0.698
No	6	90	1	
History of previous treatment				
Yes	5	31	6.94 (1.28, 37.60)	0.025
No	2	86	1	
HIV status				
Yes	2	26	1.40 (0.25, 7.64)	0.698
No	5	91	1	
History of prison				
Yes	1	17	0.98 (0.11, 8.66)	0.986
No	6	100	1	

Note that: *N* number of subjects, *OR* Odds Ratio

All the variables that were considered important were entered into the binary logistic regression models and analysis showed there were significant association between MDR-TB and history of previous anti-TB treatment (OR: 7, 95 % CI = 1. 2–37.6, *P* = 0. 025). However, there were no significant association between other variables and MDR-TB. After adjustment for interactions among the independent variables with the binary regression model; analysis also showed there were no significant association between independent variables and prevalence of MDR-TB (*P* > 0.05) with each factor other than previous treatment history.

## Discussion

The burden of MDR-TB becomes increasing in alarming pace with function of time particularly in the poorest countries. Before 20 years ago, reports showed that the prevalence of MDR-TB was almost nil or 1 % in different parts of Ethiopia [7–9]. Though, nowadays high proportion of MDR-TB were notified within the country [10, 11]. It is well understood that bacterial and environmental factors play a great role in the spread of MDR-TB. Within the population MTB, spontaneous mutation in genes responsible for drug resistance for all first line and some second line drugs, thus scenarios are highly pronounced by misuse of drugs results in rapid selection of drug resistant mutants [12].

The present study demonstrated MDR-TB is a serious issue of concern in the study area; hence the overall prevalence of MDR-TB was 5.6 % (95 % CI, 2.4–10.5 %). Which is comparable with previous reports from northwestern Ethiopia and national wide survey in Ethiopia [6, 13, 14]. On the other hand, it is lower than finding from Jimma and Bahirdar [10, 15]. The possible explanation for this difference could be due the fact that this study was conducted at the site where TB patients less likely served for medical attention and presumably they have accustomed to visit nearby and relatively advanced health institutions. Besides in previous reports the study population was presumptive MDR-TB patients, whereas in this study only smear positive TB cases were included.

Furthermore, in this finding the prevalence of MDR-TB among new and previously treated cases was respectively 2.3 % and 13.9 %. Which is consistent with previous documented data [13, 14, 16]. Many of the research findings advocated that MDR-TB are frequently identified in patients with history of TB treatment [6, 13, 14], which is also evidenced in this study. In fact, prior treatment creates opportunity for resistance MTB mutant to dominant and results challenging in the management of cases [12]. Emergence of new cases with MDR-TB has frequently related with close contact with known cases, facilitated by overcrowding [17]. Likewise, the present study showed that all of new MDR-TB cases were farmers and day laborers. The truth is a large number of people share the same house or camp for sheltering for the production of crops in the study area, which could be aggravate the issue of concern.

Moreover, this study was aimed to assess associated risk factors of acquisition of MDR-TB. History of previous treatment was the only significantly associated risk factor with MDR-TB (OR: 7, 95 % CI = 1.28–37.6, *P* = 0.02), which shows agreement with previous published reports [12, 14, 17]. This is due to the fact that prior anti-TB exposure provides only to suppress the growth of susceptible bacilli, but on the other side, it could permit suitable circumstances for the multiplication of pre-existing drug resistant mutants [12].

Even though, this study explored that history of treatment is the only risk factors for acquisition of MDR strains, however, several evidences claimed that factors including HIV/AIDS, overcrowding, smoking, opportunistic infection, lack of compliance with DOTS program, are also the potential risk factors attributes MDR-TB infection [15, 18–20]. In the recent time, global MDR-TB control programs have planned by considering the above factors, along with the highest level of compliance with guidelines (early case detection, complete treatment, administrative, environmental, or engineering controls and personal respiratory protection) [3, 12]. It is well acknowledged that DOTS strategy is the best weapon to dismantle the spread of MDR-TB [3]. Despite the fact that we have observed poor implementation of DOTS in the study area, hence it requires political commitment, sustainable budget allocation, effective drug supply and management system, and continuous monitoring and evaluation system.

## Conclusion

We report an overall prevalence of MDR-TB of 5.7 % among all cases, with the prevalence of MDR-TB among previously treated cases being 13.9 % and among new cases only 2.3 %. History of previous anti TB treatment was the only statistically significant risk factor for MDR-TB. Therefore, actions should be directed to improve the DOTS program and to maximize diagnostic laboratory facilities.

## Abbreviations

BCG: *Bacillus Calmette–Guérin*
DOTS: Directly Observed Treatment, Short-course
HIV: Human Immunodeficiency Virus
INH: Isoniazid
LJ: Lowenstein-Jensen
MDR: Multi-drug resistant
MDR-TB: Multidrug resistant tuberculosis
MTB: *M. tuberculosis*
RIF: Rifampicin
TB: Tuberculosis
WHO: World Health Organization
